# Selections

**Published:** 1891-10

**Authors:** 


### Selections.

### CHLORALAMID.

By EMORY LANPHEAR, M. A., M. D.,
Professor of Orthopœdic Surgery in the University Medical College.

### EXTRACT FROM A CLINICAL LECTURE.

This comparatively new medicinal agent is prepared by combination of two parts of chloral anhydride with one of formamide; it is found in commerce as a colorless crytalline substance, nearly tasteless, soluble in about twenty parts of water and two of alcohol. It will keep indefinitely in solution without decomposition, but cannot be dissolved in hot solutions because of chemical changes. It acts very much like chloral and sulphonal, but does not depress the heart like the former, and is much superior to the latter in that it is soluble, exerts no bad influence upon digestion, possesses no diuretic action, never causes pruritus, vertigo, diarrhœa, or other bad symptoms which sometimes follow the administration of sulphonal—in fact, experience is demonstrating the accuracy of Reichmann's observation; from chloralamid no ill effects in the circulation or in the feelings of patients are to be noted, and besides, the cost is much less than that of sulphonal. T. Lauder Brunton, in a recent report on the Relative Utility of Different Hypnotics, highly commends it, and states that with reference to certainty of action and the question of tolerance chloralamid excels.

It exerts its influence upon both the brain and spinal cord, producing sleep and reducing the motor excitement; it may be regarded as a pure hypnotic without anodyne properties, though some late reports would indicate that it has to some degree the power for partial abolition of pain. It is, then, the ideal sedative, giving prompt and satisfactory action, reliable results and absolute freedom from evil side or after effect.

Its dose is from fifteen to sixty grains. The proper method of exhibition is to give fifteen to thirty grains (according to the condition of the subject), repeating the dose in an hour if the first has not produced sleep; usually from ten to thirty grains give five to eight hours' refreshing slumber. The best method of giving it is to dissolve the required amount in about a teaspoonful of whiskey or brandy, or in a small glass of wine if the patient prefer. It may also be given in anything containing alcohol in considerable quantities, as tincture cardamon compound, tincture of hyoscyamus, etc. If for any reason it cannot be given in this manner it may be taken in powder form, and washed down with cold water or cold tea. The direction of W. Hale White, of London, is a good one, viz., tell the patient to dissolve the powder in brandy, add water to his liking, and drink it shortly before going to bed; this combination with spirits is particularly good in our surgical cases where whiskey is usually indicated, at least, in most major operations. If in any case it be better to have the medicine in liquid form, this combination may be prescribed:

℞. Chloralamid, ʒ ij.
Spts. frumenti, fl. ℥ i.
Miscē bene ut ft. solut. et adde :
Syrupi rubi ideai, fl. ℥ i.

Misce. Sig.: Dose, one tablespoonful, to be repeated in one hour if sleep is not produced. This makes a decidedly pleasant mixture of slightly acid taste and fruity aroma and flavor.

*Kansas City, Mo.*

---

ON TAKING FLUID WITH MEALS.—A great deal of misapprehension is often found to exist in the popular mind in regard to matters of eating and drinking; the cause of this, to some extent, is to be traced to old-time sayings which have come down to us in the form of a concentrated infusion of somebody's opinion upon a subject of which he or she was wofully ignorant. One of these misapprehensions to which we may refer is as to the injuriousness of taking fluid with meals. One frequently hears it laid down as a maxim that "it is bad to drink with your meals, it dilutes the gastric juice." By way of explanation we may remark that "it implies that the fluid taken is harmful." Whence this sagacious postulate originally came we cannot tell; it has quite the ring about it of an inconsequent deduction formed by a person whose presumption of knowledge was only exceeded by a lamentable ignorance of the subject. Medical men often find much difficulty in dealing with these museum specimens of antiquated science, for even educated persons are disposed to cling to the absurdities of their youth. Upon this matter Mr. Hutchinson remarks in the last number of his "Archives": "I observe with pleasure that the verdict of general experience and common sense has been confirmed by scientific experiment in the matter of taking fluid with meals. Dr. Tev. O. Stratievsky, of St. Petersburg, after elaborate trials, has found that fluids materially assist the assimilation of proteids, and announcest he following conclusion, which it is to be hoped no future experiments will controvert: "On the whole, the widely-spread custom of taking fluids during or just before one's meals, proves to be rational and fully justified on strict scientific grounds. To take fluids with the meals is almost as important an adjunct to digestion as is the mastication of solid food preparatory to swallowing it." It is obvious, however, that there is a limit to the amount of fluid one can swallow with impunity—not to speak of comfort—just as much with meals as at other times. It would be dangerous to create a general impression that fluid is good with food irrespective of quantity. It is, moreover, a well-ascertained clinical fact that an excess of cumprandial fluid does retard digestion in certain people, and gives rise to discomfort in most. A little attention to one's sensations in such matters will far better fix the desirable limit than all the "data" in the world.—*Medical Press and Circular.*

THE THERAPEUTIC VALUE OF INDIAN HEMP.—I have during the last few years been accustomed to prescribe Indian hemp in many conditions, and this drug seems to me to deserve a better repute than it has obtained. In one form of insanity, more common in women than in men, and brought on usually by mental worry, often owing to the illness of a near relative or by a moral shock, the drug acts almost as a specific. In this affection the patient is depressed and apprehensive, she imagines that animals are after her or that some one wants to injure her. There is great mental confusion and mental loss, the patient is unable to carry on any conversation, and sometimes is unable to dress herself, the condition being one of acute dementia. I have notes of several such cases that have been cured by Indian hemp within a fortnight. I usually give 10-minim doses of the tincture thrice daily, combined with iron and strychnine. I prescribe also complete rest and plenty of food. The Indian hemp is an essential factor in the treatment, for without it the rapid recovery does not ensue; it seems to remove the mental distress and the restlessness.

Indian hemp has proved very useful in my hands in the treatment of melancholia and mania. I have also found this drug of great value in the treatment of chorea when arsenic fails, as it frequently does. It may be combined with chloral with advantage in such cases. In migraine the drug is also of great value; a pill containing ¼ gr. of the extract with or without a ¼ gr. of phosphide of zinc will often immediately check an attack, and if the pill be given twice a day continuously the severity and frequency of the attacks are often diminished. I have met with patients who have been incapacitated for work from the frequency of the attacks, and who have been enabled by the use of Indian hemp to resume their employment. This drug is also a valuable gastric sedative in cases of gastric ulcer and gastrodynia. It may be combined with nitrate of silver, and it increases the efficacy of the latter. Its value is well known to asylum physicians, but it does not appear to have obtained the confidence of the profession generally. Indian hemp is also a very valuable hypnotic.—C. W. SUCKLING, M. D., *Brit. Med. Journal.*

ALCOHOL—INFLUENCE OF INEBRIETY UPON THE OFFSPRING. —A very important contribution to the alcohol question has just been furnished by Prof. Demme in Bern, in his inaugural address published by Fred Enke. The title of this substantial paper is: "The influence of alcohol on the organism of children." It is founded on personal investigations and experiences, numerous clinical observations and partly on experiments in the Pharmacological Institute of Bern. The author proves in an irrefutable manner that spirituous beverages, taken even in moderate quantities, have a much more injurious action on the juvenile organism than on the adult, and that severe disorders, especially of the nervous system, directly connected with early use of alcohol, are frequently demonstrable in children. (Diminution of longitudinal growth, promotion of rachitis, epilepsy, chorea, nervosity, cirrhotic processes, etc.) As especially injurious are pointed out alcoholic beverages given between meals, the author has ascertained repeatedly by examination of the gastric juice, whereby obstinate stomachic catarrhs with tumefaction of the abdominal lymphatic glands and increasing loss of forces are caused.

As an illustration of the fact that inebriety of parents injures enormously the vitality and health of their offspring, which is also transmitted very frequently, Prof. Demme relates the history of 10 drunken families, observed by him since 1878. Of their 57 direct descendants, 25 died in the first months of their life (general debility, eclamptic accidents, etc.); six children were idiots, five strikingly small, five epileptic, one affected with chorea and lastly idiot; five children were affected with transmitted diseases (hydrocephalus, harelip, etc.) Ten children only, i. e., 17.5 per cent were in a normal condition. On the other hand, the descendants of ten families of very moderate habits of life, observed as a comparison, were men of normal constitution, intellectually and bodily in the proportion of 81.9 per cent.—*Pacific Record.*

---

WIRING OF THE VERTEBRA.—Dr. B. E. Hadra, of Galveston, Texas, has wired the vertebra for fracture of the spine, and suggests the same operation as a means of immobilization in Pott's disease. (See *Times and Register*, May 23d.) In cases of fracture the wire sutures simply retain the fragments in close apposition ; in Pott's disease, the author thinks this method would fix the vertebra better than other methods now employed, and thus offer a better protection to the cord. It is also less annoying to the patient. The steps in the operation are as follows :

"A good long incision, the centre of which should be over the seat of fracture ; next the muscles on either side of the spinous processes should be lifted up and drawn aside with blunt instruments, but not more than to allow one to feel the contours of the bone, then a stout curved needle, armed with wire, is carried through the interspace between the spinous process of the broken vertebra and that of the next upper one, as deep as possible ; brought out, entered again into the next inferior interspace ; brought out on the other side ; entered there again into the next lower interspace ; carried around the spinous process of the vertebra, below the fracture, and again carried through the middle interspace, and, meeting the wire where it entered, well twisted together to a knot. In short, a figure-of-8 loop is carried around the spinous processes of the broken vertebra and that of the next lower one, which may be repeated as often as seems advisable. In the lumbar portion of the spine simple loops will suffice, as the processes are almost horizontal. Then the wound is closed, with or without drainage."

As yet, Dr. Hadra has operated only once, and the result has been highly satisfactory. He mentions a case of a new-born child operated upon by Dr. Wilkins, in which the last dorsal and first lumbar vertebra were separated by an interval of half an inch. The spinous processes were fixed together by the figure-of-8 suture, and the child was apparently well in a few days.

---

REMARKABLE CONTENTS OF A DERMOID CYST.—At the New York Obstetrical Society, May 5th, Dr. Mundê presented a switch of hair, five feet long, which he had removed from a dermoid cyst of a woman, 41 years of age. There was a dermoid tumor of each ovary, the right one being as large as a pregnant uterus of six months, and containing this switch of hair, the other a small ball of hair and several teeth. This large switch sprang from a small nipple-shaped protuberance at the upper portion of the sac, which was unilocular. At its root the switch was not more than an inch in diameter, gradually enlarging to a rope of matted hair as thick as the forearm. Dr. Mundê thought that probably very few cases of such enormous cranial development in a dermoid cyst are on record.—*N. Y. Journal Obstetrics.*

---

VOMITING OF PREGNANCY AND HYSTERIA.—Kaltenbach holds that the clinical history of the "cures" of cases of uncontrollable vomiting of pregnancy indicate that the disease is essentially due to hysteria. Pregnancy involves physiological and psychological conditions favorable to the development of hysterical symptoms in a modified form. Hyperemesis is often cured by a process akin to suggestion, like ordinary hysteria. "Doing something," dilatation of the os, massage, etc., often succeeds if the practitioner gains the patient's confidence, and hosts of drugs have answered, apparently, under the same conditions. On the other hand, all of these vaunted medicines and operations have failed when applied by physicians who possibly did not possess as much tact as knowledge. Hyperemesis may suddenly cease if the patient be alarmed, as in a case of the author, where the patient was reduced to a skeleton; a day being fixed for the induction of labor, her friends frightened her by saying that she could not survive such an operation. The vomiting ceased. The same sudden arrest of hyperemesis was observed by Cazeaux in a young wife whose husband was seized with symptoms of strangulated hernia. In a third case, the vomiting ceased on the outbreak of an acute exanthematous fever. Kaltenbach describes a bad case where the patient, aged twenty-one, made an unhappy marriage, and was unkindly treated by her husband. Very severe vomiting set in during her first pregnancy, and she was sent into a hospital. It was suggested to her that she had lumps of unwholesome material in her stomach, and that their removal would cure her. Some milk was given to her, ceremoniously, and shortly after the stomach was washed out. Its contents bore no indications of either over-acidity or any abnormal ferment. The patient was then informed that all was right, and that the vomiting would not return. It ceased accordingly, and she was safely delivered at term. In short, Kaltenbach urges that hyperemesis gravidarum should be treated as a purely hysterical complaint. Prompt treatment is indeed necessary, for, as in hysterical vomiting of non-pregnant women, the patient may, if at first neglected, die of syncope or nervous exhaustion, even when the vomiting has been stopped. But isolation from domestic cares and imprudent friends, with appropriate moral treatment, should be enforced before so extreme a step as artificial abortion is undertaken.—*British Medical and Surgical Journal.*

---

A RELIABLE PURGATIVE ENEMA.—The following enema has proved so reliable and satisfactory in my hands, that I feel it is worthy of a brief note:

℞. Sulphate of magnesia, 2 ounces.
Glycerin, 2 ounces.
Oil of turpentine, ½ ounce.
Water, 2 ounces.

M. Label, "To be used as an enema."

To move the bowels after abdominal section, or after plastic operations on the female pelvic organs, it has been in constant use for many months. When used alone it has moved the bowels, as a rule, promptly; and has been equally effective when given as an adjuvant to some cathartic taken by the mouth.

Prior to the employment of this formula, I had used the simple enemata of glycerin, and of glycerin and turpentine, which are distinctly inferior to the one above recommended.

The combined action of Epsom salts, turpentine, and glycerin is very effectual, not only in evacuating the rectum, but also in getting rid of flatus, which is the cause of much of the pain present after abdominal section.

I have had the opportunity, upon two occasions, of testing the activity of this enema in cases of threatened obstruction of the bowels, following operation. Other measures failing—including purgatives and large and small enemata—I have introduced a long, soft tube up the rectum, and given this enema into the descending colon, with the happiest results.

Now that the use of opium has been banished from the after treatment of cases of abdominal section, this enema has, in my hands, become the great anodyne.

When given through the rectal tube, its employment promises much in cases of partial obstruction of the bowels, also in obstruction due to paralysis of the bowel.

The enema is best given through a hard-rubber piston syringe. —*Charles P. Noble, M. D., in Medical News.*

---

TREATMENT OF PNEUMONIA WITH LARGE DOSES OF DIGITALIS.—Dr. Petresco, of Bucharest, makes an infusion of sixty grains of powdered digitalis leaves to six ounces of water, a little syrup of orange being added to sweeten it. A tablespoonful of this mixture is given every half hour. In spite of the largeness of the dose, he says he has never met with a case of poisoning, and maintains that these doses are therapeutic, and not toxic. The best results are obtained in fibrous or croupous pneumonia. The author states that if used in the way he describes, digitalis will frequently cut short an attack of true croupous pneumonia. In from twenty-four to forty-eight hours after taking the drug, a certain fall of temperature occurred, from 104° F. to 98° F., this being accompanied by a decrease in the frequency of the pulse and respiration. The digestive tract was little affected. The most marked changes were noticed in the pulse, which became slow, full, and of high tension. The conclusions to which Petresco has come are as follows :

1. When given in therapeutic doses, digitalis has a direct antiphlogistic action. 2. The dose may be raised as high as from

60 to 120 grains of the leaves given as an infusion within twenty-four hours. 3. This treatment may be continued for from two to four days, if the severity of the case requires it. 4. When improvement takes place in the circulation and respiration, this is speedily followed by a disappearance of all local signs and symptoms. 5. The success of this treatment is confirmed by the statistics. In an elaborate table of statistics, Professor Petresco shows the superiority of digitalis over the other methods. Thus the highest mortality in pneumonia (34.5 per cent.) occurred when bleeding was practiced, and the lowest (three per cent.) when tonics, alcohol, etc., were employed. When digitalis, however, was administered the mortality sank as low as 2.06 per cent. 6. From an experience of a very great number of cases, both of his own and other medical men, he maintains the doses, as given above, are perfectly harmless. 7. After a comparison of the various methods of treating pneumonia, Petresco affirms that the expectant plan is not only without reasonable foundation, but even dangerous. His experience has demonstrated that an attack of pneumonia may be aborted if the treatment is commenced at the earliest stages of this disease.—*Therapeutische Monatshefte.*

---

Life Insurance and Syphilitic "Risks."—Mr. Jonathan Hutchinson has published a paper in the *London Practitioner* on the "Modern Treatment of Syphilis," in the course of which he considers some of the more important relations of syphilis and life insurance. He states that he has recently been requested by a life insurance company to formulate a code of rules for the guidance of its examiners when considering the acceptance or rejection of applicants for insurance who have had syphilis. His advice on this subject was for the most part favorable to the applicants; with this exception, however, that he would decline those persons who, at the time of their presentation, shall be undergoing the active development of secondary symptoms. These applicants, he advises, should be told to wait until these symptoms had disappeared. He based this counsel on the fact that it is always desirable to know how well or how ill the syphilitic patient sustains the specific treatment proper to the second stage of the disease, and also how willing and attentive he may be to follow out the directions of his physician. Mr. Hutchinson holds that an insurance company might make a profitable business out of syphilitic risks accepted in the early stage of the disease and taken at the ordinary rates, for he has found that the threatened life is often a long one. In his experience such syphilitic persons appear quite as likely to attain to length of days as others who have not been syphilitic. In the cases of those who present themselves free from symptoms, but who have the history of a former attack, the advice is that they be not refused, provided that they have not definitely become the subjects of the tertiary lesions of the disease, or have not, owing to idiosyncrasy or inadequate treatment, had a prolonged siege of secondary symptoms. But even among these there are not a few who would be regarded by Mr. Hutchinson as eligible risks at ordinary rates.—*Jour. Am. Med. Association.*

---

DONOVAN'S SOLUTION IN GLEET.—The solution of the iodide of arsenic and mercury is said to be of material service in the treatment of gleet. A correspondent of the *Medical Record* feels that he is justified in calling this remedy almost a specific for gleet, so uniform has been his success with it. It should be given for this purpose, in doses of ten minims, three times daily.

---

COLD IN THE HEAD.—For this, while in the acute congestive stage, there is no better remedy than gelsemium. One good large dose, say ten minims of the fluid extract, taken before going to bed, is usually sufficient to dispose of this troublesome and uncomfortable affection.—*Medical Compend.*

---

The JOURNAL recently had a visit from Dr. J. B. S. Holmes, of Rome. The doctor has since taken a trip to New York for a look into the clinics on his special line, diseases of women. His elegant Sanitarium at Rome will be open from this date.

